# Disease-specific epigenetic deregulation of enhancers, transposons, and polycomb targets in acute promyelocytic leukemia

**DOI:** 10.1186/s13073-025-01565-y

**Published:** 2025-10-30

**Authors:** Xiangfu Zhong, Lina Cordeddu, Angelica Gamboa-Cedeno, Sofia Bengtzén, Karl Ekwall, Andreas Lennartsson, Sören Lehmann

**Affiliations:** 1 Center for Hematology and Regenerative Medicine, Department of Medicine Huddinge, Karolinska Institute, Campus Flemingsberg - NEO Building, Hälsovägen 9, SE-141 83 Huddinge, Sweden; 2 Lipid Lab Unit, Department of Medicine Huddinge, Karolinska Institute, Huddinge, Sweden; 3 Department of Medical Sciences, Hematology, Uppsala University, Uppsala, Sweden; 4 Gastroenterology and Nutrition Unit, Department of Medicine Huddinge, Karolinska Institute, Huddinge, Sweden

**Keywords:** Acute promyelocytic leukemia, Epigenetics, Chromatin state, Enhancers

## Abstract

**Background:**

Acute promyelocytic leukemia (APL) is a subtype of acute myeloid leukemia (AML), characterized by a fusion between the *PML* and *RARA* genes and by a block in the myeloid maturation at the promyelocytic stage.

**Methods:**

This study investigates the epigenetic landscape of APL by integrating ChIP-seq data on eight histone modifications and RNA-seq in APL as well as non-APL AML. APL showed a distinct chromatin profile that differed from non-APL AML.

**Results:**

We describe APL-specific changes in H3K27ac, H3K9me3, and H3K27me3 with impact on enhancer activity, repression of transposable elements, and Polycomb regulated gene repression. The APL-specific H3K27ac pattern identifies APL-specific enhancer and super-enhancer regions, including a subset of enhancers that are bound by the PML-RARA fusion protein. While chromatin bound specifically by PML-RARA were dominantly active, APL was also characterized by gain of APL-specific heterochromatin states with significant gains of H3K9me3 enriched lamina-associated domains and the transposable elements LINE, LTR, and SINE.

**Conclusion:**

These findings suggest a unique enhancer and heterochromatin profile in APL, with implications for transcription regulation and treatment response. These findings offer novel insights into the pathogenesis of APL.

**Supplementary Information:**

The online version contains supplementary material available at 10.1186/s13073-025-01565-y.

## Background

Acute promyelocytic leukemia (APL) is a subtype of acute myeloid leukemia (AML), characterized by the accumulation of promyelocytes in bone marrow. Genetically, APL is characterized by chromosomal translocation of *PML* on chromosome 15 and *RARA* on chromosome 17 that results in expression of an oncogenic fusion protein PML-RARA. Clinically, APL is characterized by a specific sensitivity to all-trans-retinoic acid (ATRA) and arsenic trioxide (ATO) which constitute the standard treatment for the disease [1]. While the initial treatment phase comes with a high risk of early death due to hemorrhages as a result of the APL specific coagulopathy, the chance of cure from the disease is very good [2, 3].

While the genetic background of APL has been well characterized, the epigenetic state of the disease, which drives the leukemogenic gene expression pattern, has been much less well defined. PML-RARA has been regarded to be a transcriptional repressor [4–7], but recently PML-RARA was shown to interact both with epigenetic repressor (*HDAC1*) and activator (*P300*) [8]. In addition, Villiers et al. have showed that PML-RARA binding may confer both activation or repression of gene expression [9]. Hence, previous results suggest that *PML-RARA* can perturb epigenetic regulation and particularly different histone modifications in a complex manner and using different mechanisms.

In this project, we set out to comprehensively characterize the epigenetic state of APL by mapping eight histone modifications associated with both transcriptional activation (H2AZ, H3K4me1, H3K4me3, H3K18ac, H3K27ac) and repression (H3K9me2, H3K9me3, H3K27me3 markers) in primary APL, AML, and normal hematopoietic progenitor cells and further integrate these ChIP-Seq data with APL’s transcriptional profile. As PML-RARA acts as a transcriptional regulator that interferes with gene expression, cellular maturation, proliferation, and apoptosis [10], the epigenetic state of PML-RARA-bound regions were specifically distinct at enhancers, super enhancers, and transposable elements. Super enhancers are clusters of enhancers closely located together that can recruit a large group of transcription factors and cofactors that affect the target genes much more extensively than typical enhancers. H3K27ac, the histone modification that is most commonly used to define active enhancers and super enhancers [11, 12], was the histone mark that was most enriched for PML-RARA-bound regions. We further identified APL-specific enhancers and super enhancers that were active in a PML-RARA-dependent manner. We could also identify APL-specific enhancers that were hijacked through the chromosomal translocation between chromosomes 15 and 17, activating genes on the other side of the chromosomal breakpoint. In general, APL was characterized by a gain of specific histone mark and where repressive histone marks were associated with transposable elements.

## Methods

### Cells from leukemia patients and healthy donors

Consecutive AML and APL patients, diagnosed at Karolinska University Hospital, as well as healthy volunteers that had approved to the study after written and oral informed consent (Ethical Review Board in Stockholm, Sweden, approval 2014/1228–31/4) were included in the study. Leukemic cells from AML and APL patients at diagnosis and healthy normal bone marrow (NBM) cells from volunteer donors were aspirated from the bone marrow and mononuclear cells were isolated by Ficoll separation. Cells were vitally frozen and analyzed upon thawing. NBM cells from the healthy donors were separated by magnetic bead sorting of CD34 + cells before analysis. There were 5 APL, 9 non-APL AML, and cells from 5 healthy donors included in the study. All APL and AML samples underwent ChIP-seq for all 8 histone marks, RNA-sequencing, and panel DNA sequencing to detect disease-specific mutations. For NBM samples, each ChIP-seq analysis was performed in 3 to 4 donors. One APL patient with sufficient number of cells also underwent HiC.

### Chromatin immunoprecipitation followed by sequencing (ChIP-Seq)

Bone marrow samples from patients and healthy donors were prepared and chromatin immunoprecipitated, using the iDEAL ChiP-seq kit Diagenode (C01010051). The following antibodies were used for the immunoprecipitation: H2AZ (Abcam, Ab 4174), H3K4me1 (Abcam, Ab 8895), H3K4me3 (Diagenode, C15410003), H3K9me2 (Abcam, Ab 1220), H3K9me3 (Abcam, Ab 8898), H3K18ac (Diagenode, C15410139), H3K27ac (Abcam, Ab 4729), and H3K27me3 (Diagenode, C15410069). Libraries were prepared using NEB Ultra II regents, then sequenced on Illumina HiSeq 2000 or NextSeq 550. The sequencing was performed at the Bioinformatics and Expression Analysis core facility, Karolinska Institute.

### RNA-sequencing on primary patient samples

Pair-end sequencing of total RNA samples on Illumina HiSeq 2500 with HiSeq SBS Kit v4, at Eurofins Genomics GmbH, processed by HiSeq Control Software 2.2.58, RTA 1.18.64 and bcl2fastq 1.8.4.

### APL cell line and cell culture

NB4 cell line was cultivated with RPMI (Thermo Fisher Scientific CAT# 11554526), Fetal Bovine Serum 10% (Fisher Scientific CAT# 11550356), and PenStrep (Gibco CAT# 15140-122), at 37 °C, CO_2_ 5%, and 95% of humidity.

### RNA-sequencing on NB4 cells

The RNAs extracted from NB4 cells after being treated with ATRA 1 µM, DMSO 0.01% as control for 72 h, using the AllPrep DNA/RNA Mini Kit (Qiagen CAT# 80204). Total RNA was subjected to quality control with Agilent Tapestation according to the manufacturer’s instructions. To construct libraries suitable for Illumina sequencing, the Illumina stranded mRNA prep ligation sample preparation protocol was used with starting concentration between 25–1000 ng total RNA. The protocol includes mRNA isolation, cDNA synthesis, ligation of adapters, and amplification of indexed libraries. The yield and quality of the amplified libraries was analyzed using Qubit by Thermo Fisher and quality was checked by using Agilent Tapestation. The indexed cDNA libraries were normalized and combined, and the pools were sequenced on the Illumina Nextseq 2000 P2 100 cycles kit sequencing run, generating 58 base paired end reads with dual index 10 + 10 base pairs. Basecalling and demultiplexing was performed using bcl2fastq (v2.20.0) software with default settings generating Fastq files for further downstream mapping and analysis.

### ChIP-Seq data processing and analysis

The data was processed by implementing the nf-core chipseq pipeline (v1.2.1) [13]. Raw reads were mapped to human reference genome hg19 using bwa (v0.7.17-r1188), duplicated reads were removed afterwards. The bigwigs files were generated by deeptools (v3.4.3) [14] and RPKM normalized. ChIP-seq peaks were called by MACS2 (v2.2.7.1) [15, 16], and differential modified peaks were identified by DiffBind [17, 18], which normalized with TMM with library size in considering. ChIP-seq density plots and heatmaps were plotted by deepTools (v3.4.3) [14]. The gencode.v39lift37.annotation.gtf was used as the reference genome annotation, same as in RNA-Seq data analysis.

The bigwig files are generated by deepTools [14] for visualization on the Integrative Genome Viewer (IGV) [19–21], merging bam files from each biological groups, sorting and indexing using samtools, and the scaling using deepTools.

### Chromatin state characterization

ChromHMM [22] was used to identify chromatin state patterns based on eight histone modifications: H2AZ, H3K4me1, H3K4me3, H3K9me2, H3K9me3, H3K18ac, H3K27ac, and H3K27me3. Bam files were converted to bed files, then binarized the bed files using the BinarizedBed command from chromHMM, at 1 kb resolution. ChromHMM were used to learn models based on 10 to 20 chromatin states. We selected the model of 15 chromatin states generated by chromHMM, based on correlations between models (10 states model to 20 states model).

Chromatin states annotation was made on chromatin states with the help of using the data from the Genome Browser annotation track database (https://genome.ucsc.edu/cgi-bin/hgTables) for hg19/GRCh37: conservedTFBS, CpGislands, NKI_LADs, RepeatMasker, SimpleRepeats, UCSCknownGenes, and UCSCknownGenes_TSS.

### Enhancer and super enhancer characterization

We identified the enhancers and super-enhancers by the ROSE (rank order of super enhancers) [23] using -t 500 (-t TSS zone exclusion distance) based on the ChIP-Seq data of H3K27ac. DiffBind was used for differential analysis of histone modification ChIP-seq data. The AME (Analysis of Motif Enrichment) [24] part of the MEME tool suite was used to identify sequence motifs from query enhancers.

PML-RARA target sites were retrieved from Tan et al. [8]. They identified 6415 PML/RARA direct targets on the NB4 cell line, by ChIP-seq data using the antibody against the PML/RARα fusion site. ChIA-PET data of RNAPII was retrieved from GEO on NB4 cell line (accession number GSE137661) [25]. The 2D contact map files (hic files) used Juicer tools [26] and were visualized using IGV browser.

### ClinSeq cohort

More than 300 AML patients were included in the ClinSeq cohort as previously described [27]. We used 144 normal karyotype AML patients and 13 APL patients from the ClinSeq cohort for gene expression validation.

### RNA-Seq data processing

Bulk RNA-seq data was processed by the nf-core rnaseq pipeline (v1.4.2) [13]. In detail, the raw sequencing data were processed by the analysis pipeline nfcore rnaseq 1.4.2. It used FastQC (v0.11.8) [28] and MultiQC (v1.7) [29] for quality control; Trim Galore!(v0.6.4) [30] for adapter trimming; STAR (vSTAR_2.6.1d) [31] for mapping reads to the human reference genome hg19; featureCounts (v1.6.4) [32] gene level quantification. The human reference genome annotation GENCODEv39 (gencode.v39lift37.annotation.gtf) was used for gene expression quantification. Differential expression analysis was implemented by DESeq2 [33].

### HiC sequencing 

The sequencing libraries of the patient primary samples were constructed using Dovetail OmniC at the National Genomics Infrastructure, Stockholm. The libraries were sequenced on Illumina NovaSeq 6000 (NovaSeq Control Software 1.8.1/RTA v3.4.4) with a 151nt(Read1)−19nt(Index1)−10nt(Index2)−151nt(Read2) setup using “NovaSeqXp” workflow in “S4” mode flowcell. The Bcl to FastQ conversion was performed using bcl2fastq_v2.20.0.422 from the CASAVA software suite. The quality scale used is Sanger/phred33/Illumina 1.8 +. The HiC data then analyzed with the nfcore hic (v2.1.0) pipeline.

### Statistical analysis

R (version 4.1.1) was used for statistical analysis and graphics. Fisher’s exact test and Welch Two Samples *t*-test were used in R.

## Results

### Patient characteristics

**Fig. 1:**
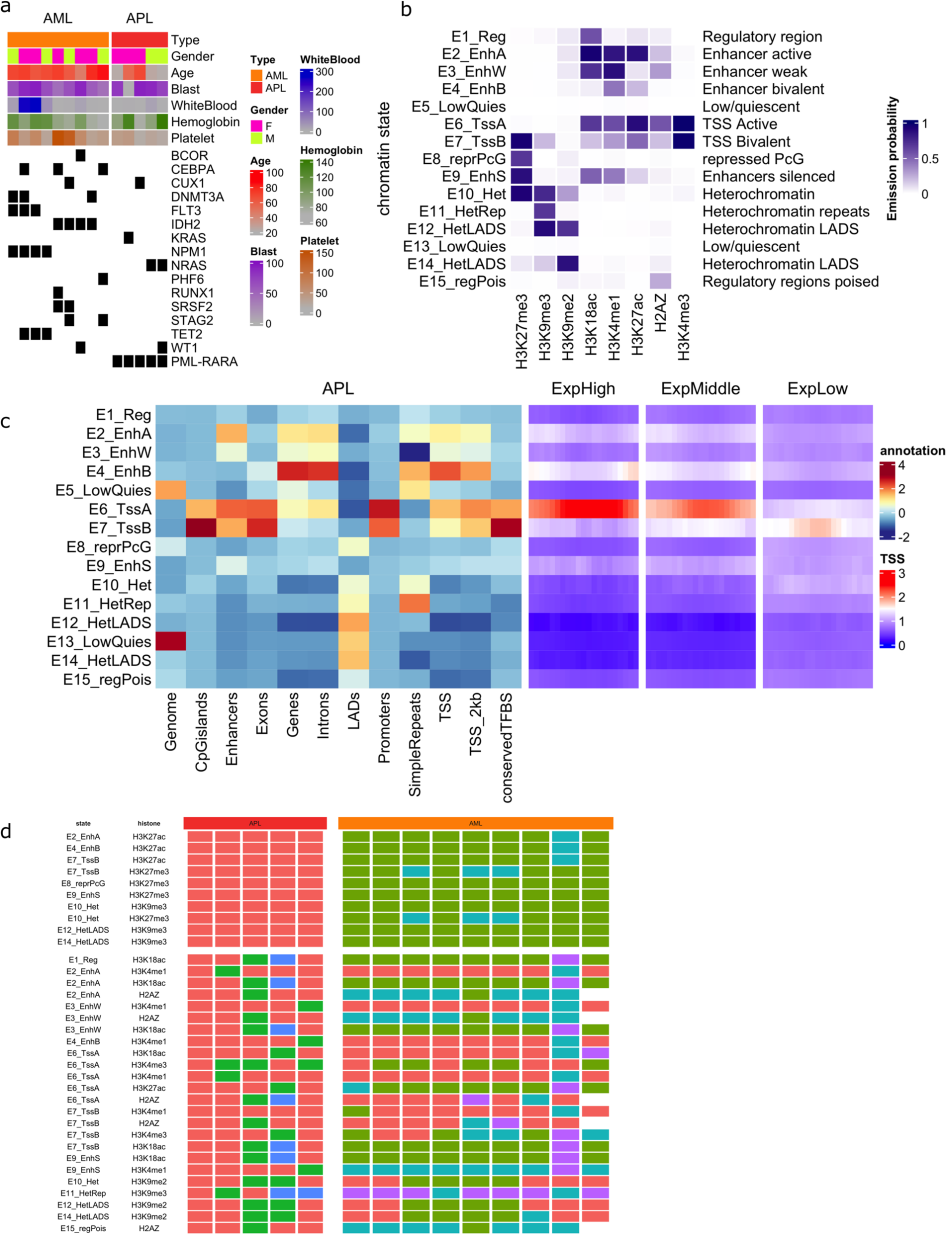
Characterization of patients and chromatin states in APL.** a** Mutational, laboratory, and clinical characteristics of APL and non-APL AML patients (from now referred to as AML) included in the study. **b** Dynamic pattern of 15 chromatin states based on integrated ChIP-seq on H2AZ, H3K4me1, H3K4me3, H3K9me2, H3K9me3, H3K18ac, H3K27ac, and H3K27me from patient cells. **c** Functional genomic annotation enrichment using publicly available data for the APL 15 chromatin states (left panel). Enrichment of gene expression data from RNA-seq of the same primary patient samples (right panel). Genes are grouped into high, middle, and low expression based on their RPKM. **d** Heatmap representing results from 34 unsupervised consensus clusterings for each histone marker within each relevant chromatin state (APL left, AML right, individual clusterings are shown in Fig. S2). The heatmap colors (red, blue, turquoise, purple, green) represent the different clusters (*n* = 2–5) from each individual clustering in Fig. S2. The top of the heatmap lists all combination of histone marks and chromatin states where the clustering displayed no overlapping samples between APL and AML, thus identifying disease-specific histone marks/chromatin states

To characterize the APL-specific epigenetic landscape, 5 APL patients, 9 normal karyotype non-APL AML (from now on referred to as AML in the manuscript) patients, and CD34 + bone marrow samples from 5 healthy donors were analyzed. The mutational and the clinical profile of the patients are shown in Fig. 1a. The mutational profiles of the AML patients were more heterogeneous compared to the APL patients (Fig. 1a). In APL patients, mutations in addition to the PML-RARA fusion included *NRAS* mutations in two cases and mutations of *KRAS*, *CUX1*, and *ASXL1* in one case each. The details of the mutations as well as the karyotypes of the APL patients are presented in Table S1.

### Chromatin states define the epigenetic landscape of APL

A comprehensive epigenetic characterization was performed, by ChIP-Seq profiling of eight histone modifications in all samples including H2AZ, H3K4me1, H3K4me3, H3K9me2, H3K9me3, H3K18ac, H3K27ac, and H3K27me3. In addition, bulk RNA-seq was performed on all cases. Based on the ChIP-seq data, a 15-chromatin state model was built to characterize the epigenetic landscape of APL (Fig. 1b).

Functional epigenetic and transcriptomic characteristics were annotated for each of the 15 chromatin states, e.g., describing the states as transcriptionally active or silent, as being related to enhancers, transcription start sites (TSSs), or lamina-associated domains (LADs), etc. (Fig. 1b). To ensure the quality of the chromatin state annotation, the fold enrichment of each state in a set of publicly available genomic annotations was investigated (Fig. 1c). This analysis confirmed the expected associations for each state such as with CpG islands, promoters, exons, TSSs, or enhancers. To further confirm the expression profiles representing the 15 chromatin states, RNA-seq expression data from the same patients were aligned to each state. An RPKM greater than 2 was classified as high expression while an RPKM less than 0.01 was classified as low expression, and median RPKM value was established for expression levels between 0.01 and 2 (Fig. S1a). The TSSs were extracted to perform the neighborhood enrichment (Fig. 1b right panel). The TSSs of highly expressed genes were enriched in TSS active state, whereas the intermediately expressed genes decreased their enrichment in TSS active state and increased enrichment in TSS bivalent state while the lowly expressed genes further significantly decreased in the TSS active state. Hence, our chromatin state model and annotation confirmed the relationship between the epigenome and the transcriptome of APL. The genomic annotation enrichment in each chromatin state was similar between APL and AML cases (Fig. 1c and Fig. S1c).

### Specific histone marks in enhancer, bivalent, and repressive chromatin states separate APL from AML

In order to find the histone marks that best defined APL and separated APL from AML, unsupervised consensus clustering using the top 2000 most variable peaks of all 8 histone marks within each chromatin state on all APL and AML samples was performed. A complete display of the clusterings of all the 34 combinations (*k* = 2 to *k* = 5 clusters) is shown in Fig. S2. The same clustering approach was also applied using up to the 2001–4000th most variable features (Fig. S3) which did not improve the clustering compared to the top 2000 most variable peaks, suggesting that the generated data best captured the biological differences between APL and AML. Figure 1d shows the results of all the 34 clusterings and three histone marks within eight specified chromatin states could completely separate APL from AML with no overlap between APL and AML samples (Fig. 1d). Among the active histone marks, H3K27ac was the most specific for APL and completely separated APL from AML cases in enhancer (E2 and E4) and bivalent TSS (E7) chromatin states. In contrast, clustering of other active histone marks such as H3K4me1 and H3K4me3 showed overlap between APL and AML (Fig. 1d). Unsupervised consensus clustering of the 2000 most variable chromatin regions from the H3K27ac ChIP-seq data in the active enhancer state (E2) confirmed the separation between APL and AML (Fig. S4a). Also, the repressive mark H3K9me3 within heterochromatin states E10, E12, and E14 completely separated APL from AML as did H3K27me3 within states E7, E8, E9, and E10 (bivalent TSS, repressed Polycomb Group (PcG), silent enhancer, and heterochromatin, respectively) (Fig. 1d). In general, H3K27ac and H3K27me3 separated APL from AML to a high degree, regardless of chromatin state. In addition, we also calculated the state-pairwise Jaccard index of each chromatin state between APL and AML which confirmed that enhancer states and heterochromatin states show less correlation between APL and AML compared to the other states such as TSS associated states that display a higher correlation (Fig. S1c). This further demonstrates that enhancer states (E2, E3, E4, E9) and heterochromatin states (E10, E11, E12, E14) are more different in APL compared to AML, while TSS associated states are more similar. As chromatin states related to enhancers and heterochromatin best could distinguish APL from AML, we further studied the differential histone modifications, and the gene expression patterns related to these epigenetic states.

All-trans retinoic acid (ATRA) is used as the primary induction therapy for newly diagnosed APL patients inducing myeloid differentiation in APL cells. To explore the basal epigenetic status of ATRA responsive genes, we performed RNA-seq on NB4 cells treated with ATRA (1 µM for 72 h) and identified ATRA responsive genes as significantly differentially expressed genes (p.adj < 0.05 and | log2FoldChange|> 2, 565 down- and 923 upregulated genes). Among these genes, 302 (of 719 ATRA upregulated) and 192 (of 651 ATRA downregulated) genes overlapped with PML-RARA binding sites from Tan et al. [8] defined as described below. The enrichment of both up- and downregulated genes for PML-RARA targeted genes were significant by hypergeometric test (*p*-value 1.2e − 158 and 5.1e − 70, respectively). The TSS regions of ATRA responsive (upregulated and repressed) genes were more enriched in bivalent chromatin states (Fig. S1d).

### Active histone modifications are enriched at PML-RARA binding sites

**Fig. 2:**
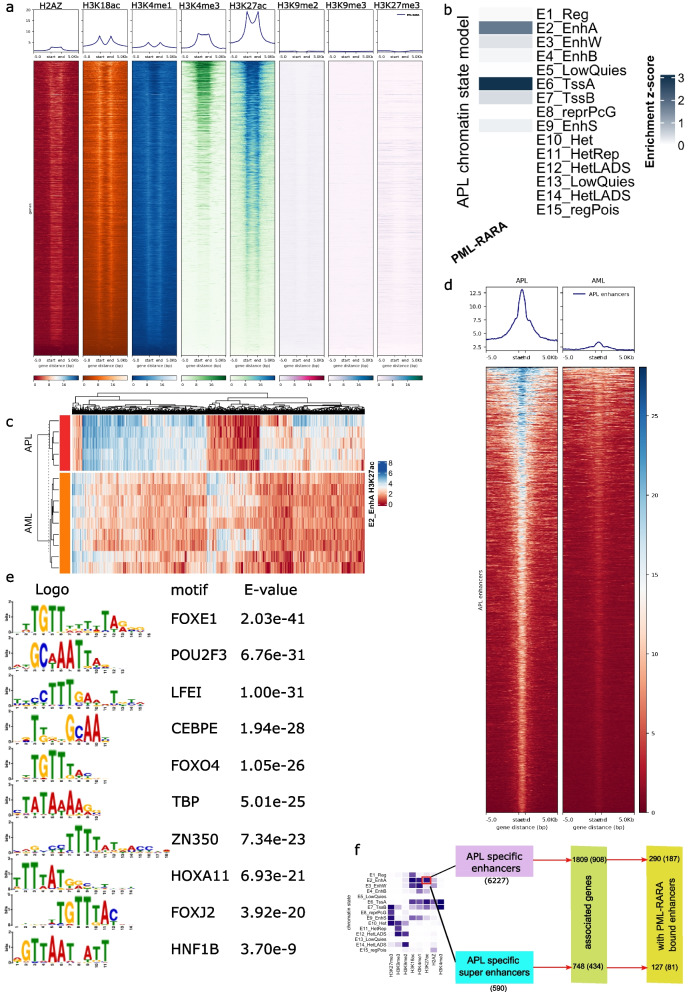
Enhancer profiles differ between APL and AML.** a** Heatmaps and density plots of histone modifications within PML-RARA target sites (defined by Tan et al. [5]). **b** Enrichment of PML-RARA target sites in the 15 APL chromatin states. **c** Heatmap of most variable H3K27ac regions between APL and AML samples. **d** Heatmap of H3K27ac for APL-specific enhancers in APL and AML samples. **e** Top 10 enriched motifs from APL-specific enhancers. **f** Flowchart for the analysis of enhancers, super enhancers, and integration of RNA-seq data. A total of 6227 APL-specific enhancers and 590 APL-specific super enhancers were identified. From these, 1809 and 748 genes were significantly correlated with APL-specific enhancers and super enhancers, respectively. Furthermore, 290 and 127 genes showed significant correlation with APL-specific enhancers and super enhancers that are bound by PML-RARA. The numbers in parentheses indicate genes that are differentially expressed in APL patient cells

In order to relate the epigenetic states to sites targeted by the APL-specific PML-RARA fusion protein, we retrieved the PML-RARA binding sites identified by ChIP-seq by Tan et al. [8] and integrated with our epigenetic and transcriptomic analysis. Integrating the histones pattern with PML-RARA binding sites revealed a pattern with low binding at repressive marks (H3K9me2, H3K9me3, and H3K27me3) and a prominent binding at active histone marks (H3K4me3, H3K27ac, H3K18ac) (Fig. 2a). Among them, H3K27ac showed the most frequent histone modification within PML-RARA binding sites, and given that this is the fusion protein that drives APL leukemogenesis, it is also in line with the separation of APL and AML by H3K27ac as shown in Fig. 1d. This contrasts to the widespread notion of PML-RARA fusion protein as a dominant negative repressor [4–7]. Half of PML-RARA binding sites (49%, 3007 of 6551) were located within or close to TSS regions (within 3 kb, Fig. S4b). Furthermore, relating PML-RARA binding sites to the chromatin states, PML-RARA binding sites were specifically enriched in active enhancer (E2) and TSS (E6) chromatin states (Fig. 2b), and to a lesser degree in bivalent TSS (E7) and weak, bivalent and silenced enhancer (E3, E4, and E9) states (Fig. 2b).

### APL presents with specific enhancer and super enhancer profiles

As H3K27ac at enhancers specifically separated APL from AML and as PML-RARA binding sites correlated best with H3K27ac (Fig. 1d, Fig. S4a, Fig. 2a), we next characterized the epigenetic status of APL-specific enhancer states using H3K27ac ChIP-seq data. To define the APL-specific enhancers, differential analysis was performed on H3K27ac modification sites within the active enhancer chromatin state E2 in APL versus AML samples. A heat map of the top 2000 most differentially modified H3K27ac peaks display a mostly homogenous pattern in APL enhancers (Fig. 2c). The differential analysis of E2 active enhancer chromatin states identified 6227 significantly gained regions as APL-specific enhancers (p.adj < 0.05 and log2FoldChange > = 1) with a distance to closest TSS of more than 500 bp. The clear enrichment of the H3K27ac ChIP-seq signal at enhancer regions in APL compared to AML enhancers is shown in a signal intensity plot in Fig. 2d for APL-specific enhancers and in Fig. S4c for AML-specific enhancers.

Motif analysis of the APL-specific enhancers identified 108 TFs (Table S2) where the top 10 are displayed Fig. 2e. GO analysis showed these 108 TF genes were related with myeloid differentiation (Fig. S4d, for the complete results of the GO analysis, see Table S3). Many TFs (*CEBPE *[34, 35], *LEF1 *[36–38], *FOXO4 *[39, 40], *TBP* [41]) have well known functional roles in hematopoietic differentiation [42, 43].

Next, we identified genes potentially regulated by the APL-specific enhancers by “guilt-by-association” [44] approach, which calculates the correlation between each enhancer and the expression of genes less than 1 Mb up- or downstream of the enhancer, using a workflow described in Fig. 2f. Genes with an expression that significantly correlated (*r* > = 0.7, *p*-value < = 0.01) with the H3K27ac ChIP-seq signal of the enhancers were used in the downstream and enrichment analyses. Through this analysis, significantly correlated enhancer-gene pairs were found between 2273 APL-specific enhancers and 908 differentially expressed genes in APL (Table S4). Half of the APL-specific enhancers correlated genes that were significantly differentially expressed in APL by RNA-seq (Fig. S4f). Based on these genes, the biological difference between APL and AML was defined by gene enrichment of KEGG, HALLMARK, and REACTOME (Fig. S4e and Table S5). In the HALLMARK sets, “Estrogen Response Late” and “P53 Pathway” were identified only in APL while “Kras Signaling Dn” and “Notch Signaling” were only found in AML. Estrogen has shown to be required for normal hematopoiesis [45], and estrogen receptor pathway has been shown to be a potential treatment target in AML cells [46].

With the aim to define enhancers that are targeted directly by the PML-RARA fusion protein, we identified 307 out of 6227 the APL-specific enhancers that co-localized with PML-RARA binding sites. Within these 307 APL-specific enhancers bound by PML-RARA, there were 290 genes where the gene expression correlated with H3K27ac signal of these enhancer regions (*r* > = 0.7, *p*-value < = 0.01). Of the 290 genes, 187 were also differentially expressed (p.adj < 0.05) in APL compared to AML. REACTOME pathways and Gene Ontology analysis of these genes are shown in Fig. S4e. Among these differentially expressed genes specifically linked to PML-RARA bound active APL-specific enhancers, were genes involved in myeloid differentiation and leukemogenesis, for example *CEBPE*,* COL21A1*, and *PPARG*. *CEBPE* is a critical transcription factor in myeloid cell differentiation [34] and leukemogenesis [35]. PPARG expression is associated with resistance to *PPARG agonist* pioglitazone which has shown antileukemic effect [47]. Among the genes that were upregulated in APL and linked to PML-RARA bound APL enhancers, 54 were also among the ATRA responsive genes that changed expression in response to ATRA in NB4 cells (Fig. S4g). These genes associated with PML-RARA bound enhancers were mostly downregulated in ATRA-treated NB4 cells, especially in response to longer ATRA exposures (72 h and 120 h) (Fig. S4h). Among these genes were *UNCX* which is a transcription factor that has been linked to AML and particular APL [48]. Elevated *UNCX* expression has been suggested to cause a differentiation arrest in myeloid cells [48].

**Fig. 3:**
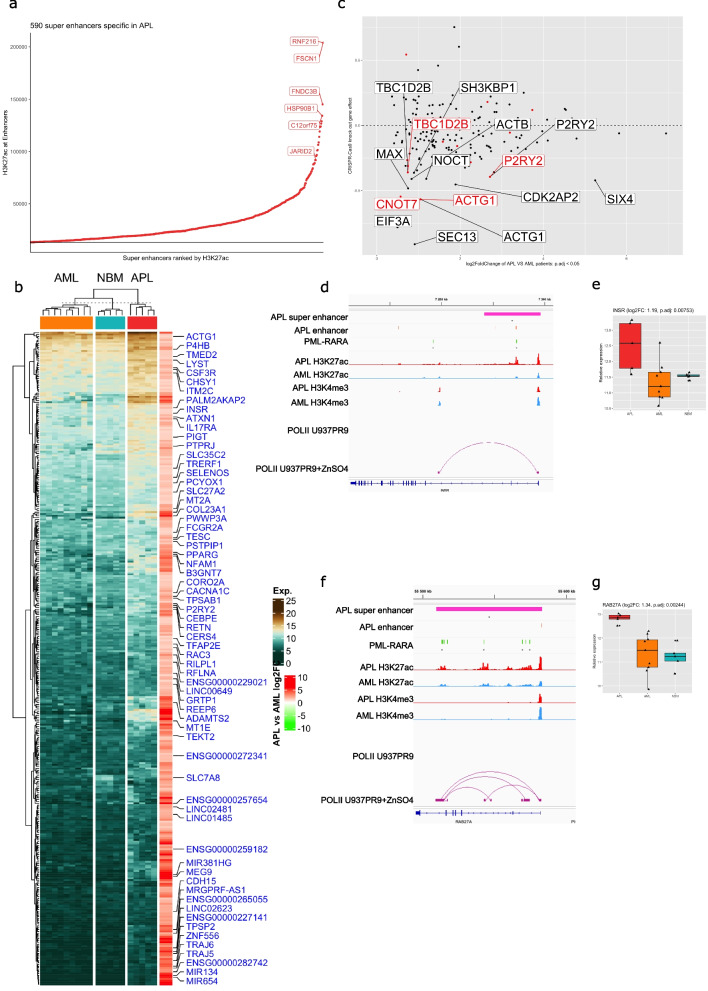
APL-specific super enhancers and their gene expression regulation. **a** Ranked scatter plot of APL-specific super enhancers. Genes known to be associated to leukemogenesis that are linked to the top 20 APL-specific super enhancers are shown in red. **b** Expression heatmap of the genes that correlated with APL-specific super enhancers (*p*-value < 0.01, *r* > = 0.7). Genes that overlap with PML-RARA binding are marked in blue. **c** Scatter plot of APL super enhancers associated genes based on gene dependency data from genome-wide CRISPR knockout screens in NB4 cells from DepMap. *X*-axis shows log2FoldChange of differential expression analysis in APL vs. AML patient cells (p.adj < 0.05) and *Y*-axis shows the degree and type of gene effect by the CRISPR knockout screens in NB4 cells, data retrieved from DepMap. The top 15 genes based on highest gene dependency (negative gene effect score) are annotated with gene symbols. Gene symbols in red color are associated with super enhancers bound by PML-RARA. **d** Integrative Genomics Viewer (IGV) track of *INSR* shows tracks (from the top to bottom) APL-specific super enhancers, APL-specific enhancers, PML-RARA binding sites, H3K27ac profile in APL, H3K27ac profile in AML, H3K4me3 profile in APL, H3K4me3 profile in AML, RNAPOLII loops by ChIA-PET in U937 cells (without PML-RARA expression), RNAPOLII loops by ChIA-PET in U937 + ZnSO_4_ cells (with PML-RARA expression). **e** Box plot of relative *INSR* expression in APL and AML patient cells and normal CD34^+^ bone marrow cells. **f** IGV track of *RAB27A* showing (from top to bottom) APL-specific super enhancers, APL-specific enhancers, PML-RARA binding sites, H3K27ac profile in APL, H3K27ac profile in AML, H3K4me3 profile in APL, H3K4me3 profile in AML, RNAPOLII loops by ChIA-PET in U937 cells (without PML-RARA expression), RNAPOLII loops by ChIA-PET in U937 + ZnSO_4_ cells (with PML-RARA expression). **g** Box plot of relative *RAB27A* expression in APL AML patient cells and normal CD34^+^ bone marrow cells

Super enhancers have been suggested to play an important role in cancer development [49–53]. Therefore, we further characterized the profile of super enhancers specific to APL as identified by ROSE, using the active enhancer state E2 segments as enhancer peaks. Through this approach, 1415 super enhancers were identified in APL and 1399 in AML. By intersecting them, 590 APL unique super enhancer were identified that contained at least one APL-specific enhancer. Several of the top 20 APL super enhancers are linked to genes known to have functional roles in leukemia, for example *RNF216*, *FSCN1*, *FNDC3B*, *HSP90B1*, *C12orf75*, and *JARID2* (Fig. 3a), among them, *JARID2* is a hematopoietic tumor suppressor through recruiting *PRC2* to repress self-renewal pathways [54]. Furthermore, out of 748 genes significantly correlated (*p*-value < 0.01, *r* > = 0.7) with APL-specific super enhancers were 434 genes also differentially expressed in APL vs. AML patient samples (p.adj < 0.05). To identify APL-specific super enhancers that also are bound by the PML-RARA fusion protein, we identified 71 super enhancers that were bound by PML-RARA. Those 71 super enhancers were associated with 127 genes, whereof 81 were significantly upregulated in APL (Fig. 3b and Table S6).

In order to validate the potential functional role of these super enhancers, we cross-analyzed all APL super enhancer-associated genes with the Dependency Map (DepMap) dataset for the APL NB4 cell line also harboring *PML-RARA*. The DepMap dataset is based on genome-wide CRISPR-Cas9 and RNAi screens to identify genes essential for the growth and survival of specific cancer cell types. Through this cross-analysis, we show that NB4 cells are highly dependent on 134 out of 748 APL-specific super enhancer-associated genes, suggesting that the APL-specific super enhancers regulate genes important for growth and survival of APL cells (Fig. 3c). Among the genes showing the top dependency scores, four genes were associated with APL-specific super enhancers bound with PML-RARA and they were also upregulated in APL: *CNOT7*, *ACTG1*, *P2RY2*, and *TBC1D2B*. *CNOT7* has reported to be involved in cell proliferation [55] and *ACTG1* is a cell adhesion gene, reported to be downregulated in KMT2A::MLLT10 AML patients [56] and potentially affecting leukemia progression. Finally, activation of P2RY2-AKT signaling has been shown to promote leukemogenesis in acute myeloid leukemia [57].

### PML-RARA binding at APL-specific super enhancers change super enhancer interaction patterns

An APL-specific super enhancer was identified in the vicinity of *INSR*, the insulin receptor, containing multiple *PML-RARA* binding sites (Fig. 3d). Using available chromatin interaction data by ChIA-PET [25] in an inducible *PML-RARA* model in U937 cells, we showed that genomic interactions within *INSR* the gene were only present when *PML-RARA* was induced and absent without the *PML-RARA* fusion, suggesting that *PML-RARA* binding modifies the super enhancer interactions. The PML-RARA mediated super enhancer and *INSR* interaction correlated with induced expression of *INSR* in APL compared to both AML and normal bone marrow (Fig. 3e). The *INSR* expression does not change during normal myeloid differentiation (Fig. S5a), suggesting that the increased expression is PML-RARA driven and not a reflection of the normal maturation state of promyelocytes. In agreement with PML-RARA-induced *INSR* expression, *INSR* is downregulated in NB4 cells upon ATRA-induced differentiation in GSE131325 [58] and this study (p.adj = 0.0003, log2FoldChange = − 1.5) (Fig. S5b and c). The genes associated with PML-RARA-bound super enhancers are predominantly downregulated in response to ATRA exposure, particularly during longer ATRA incubation (72 h and 120 h) (Fig. S5d).

Similarly, *RAB27A* and *S100P* are two genes linked to tumor development [59–62] that are located within APL-specific enhancers that also harbor multiple PML-RARA binding sites and where enhancer interactions are dependent on the PML-RARA protein (Fig. S5e). The expression of these genes is also highly associated with the activity of the associated enhancers (*RAB27A*: *r* = 0.76, *p*-value = 0.0017; *S100P*: *r* = 0.84, *p*-value = 0.00015). Expression patterns and gene interactions are shown for *RAB27A* in Fig. 3f, g and for *S100P* in Fig. S5f-g. In conclusion, PML-RARA binds at APL-specific super enhancers and changes the interaction patterns, associated with enhanced gene expression of the corresponding genes such as *INSR*, *RAB27A*, and *S100P*.

### Enhancer hijacking at the chr15 and chr17 break point increases ZNF385C expression

**Fig. 4:**
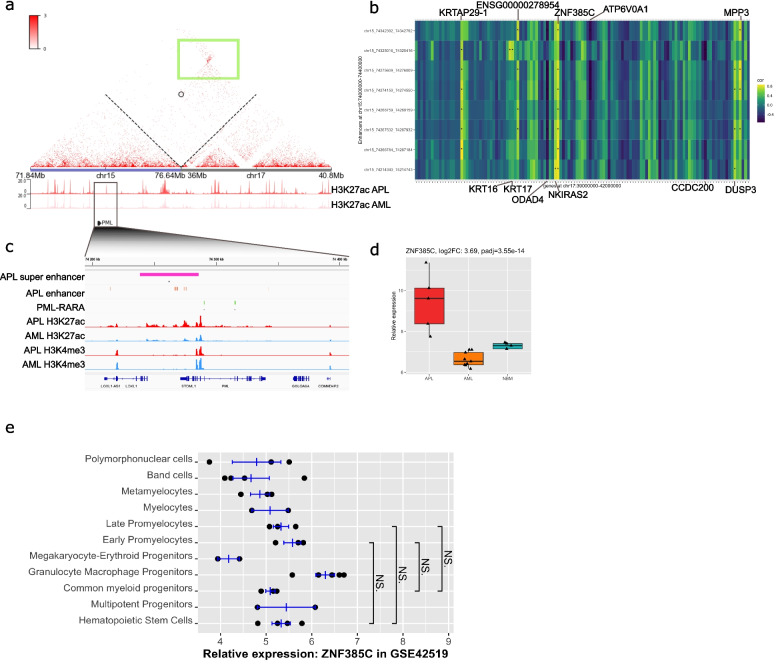
Hijacked enhancers and super enhancers deregulate gene expression.** a** Heatmap of HiC data around the t(15;17) chromosome translocation (indicated by the black cycle) in an APL patient. H3K27ac tracks (APL top (red), AML bottom (pink)) are shown below the HiC heatmap, blue line represents chromosome 15, and gray line for chromosome 17. Green box highlights interactions at loci between chr15 and chr17 over the *PML-RARA* fusion point. **b** Heatmap of correlation between enhancer activities (by H3K27ac) at chr15 and expression of genes at chr17. Star (*) represents a *p*-value below 0.05; color represents the correlation coefficient. **c** IGV track of zoomed region at chr15. Tracks (top to bottom) show super enhancers in APL, enhancers in APL, PML-RARA binding sites, H3K27ac in APL, H3K27ac in AML, H3K4me3 in APL, H3K4me3 in AML. **d** Box plot of *ZNF385C* gene expression in APL and AML patient cells and normal CD34^+^ bone marrow cells. *ZNF385C* is upregulated in APL compared to AML by a log2FoldChange of 3.69. **e** Scatter plot of *ZNF385C* expression during normal hematopoietic differentiation (GSE42519), *t*-test performed for comparing early and late promyelocytes with hematopoietic stem cells. *T*-tests show that *ZNF385C* expression does not differ between early and late promyelocytes compared to hematopoietic stem cells

To investigate potential new chromatin interactions between APL-specific super enhancers on one side of the breakpoint of the 15–17 chromosome translocation with genes on the other side of the break point, HiC sequencing was performed in one APL and AML patient. In the APL patient sample, the chromosome translocation was confirmed by the HiC data (Fig. 4a). When overlapping HiC data with H3K27ac ChIP-seq data, a set of H3K27ac peaks (with APL-specific enhancers) around the *PML* gene were found in the APL sample, suggesting the presence of enhancers and super enhancers (Fig. 4a). HiC data showed interactions between these loci over the fusion point between chr15 and chr17. To look for potential interactions between these enhancers and genes, we calculated the Pearson correlation between enhancer H3K27ac levels and gene expression around the breakpoint region of chr15 and chr17. Analysis of correlations between enhancers on chr15 and genes on chr17 revealed several significant correlations (Fig. 4b), while for enhancers at chr17 paired with genes at chr15, no significant correlation could be observed (Fig. S5h). The expression of *ZNF385C* (Zinc finger protein 385C) on chr17 showed significant correlation with 7 enhancers on chr15 (Fig. 4b). *ZNF385C* has been associated with the late stages of leukemia tumor progression [63], and regulates gene transcription in a *p53*-dependent manner [64]. An APL-specific super enhancer, covering 5 APL-specific enhancers, was found in the vicinity of the *PML* gene locus (Fig. 4c). The expression of *ZNF385C* was upregulated in APL compared to AML cells in a validation cohort consisting of 144 AML and 13 APL patients (Fig. 4d, Fig. S5i). Moreover, the *ZNF385C* expression did not change during normal myelopoiesis, suggesting that the upregulation is specific to APL cells and not due to the differentiation stages (Fig. 4e). These data suggest that the upregulation of *ZNF385C* might be due to that enhancers on chr15 are hijacked by genes on chr17, which may contribute to leukemogenesis in APL.

### The H3K9me3 profile distinguishes APL from AML

To systematically investigate heterochromatin states that distinguished APL from AML, we further studied the 3 heterochromatin-related states E10, E12, and E14, where the two latter are related to lamina-associated domains (LADs) (Fig. 1b and d). LADs are involved in the chromosome architecture and represent heterochromatin adjacent to the nuclear lamina with silenced gene repression [65, 66]. As H3K9me3 completely separated APL from AML in all three heterochromatin states, we focused on the H3K9me3 mark when defining the APL-specific heterochromatin regions.

**Fig. 5:**
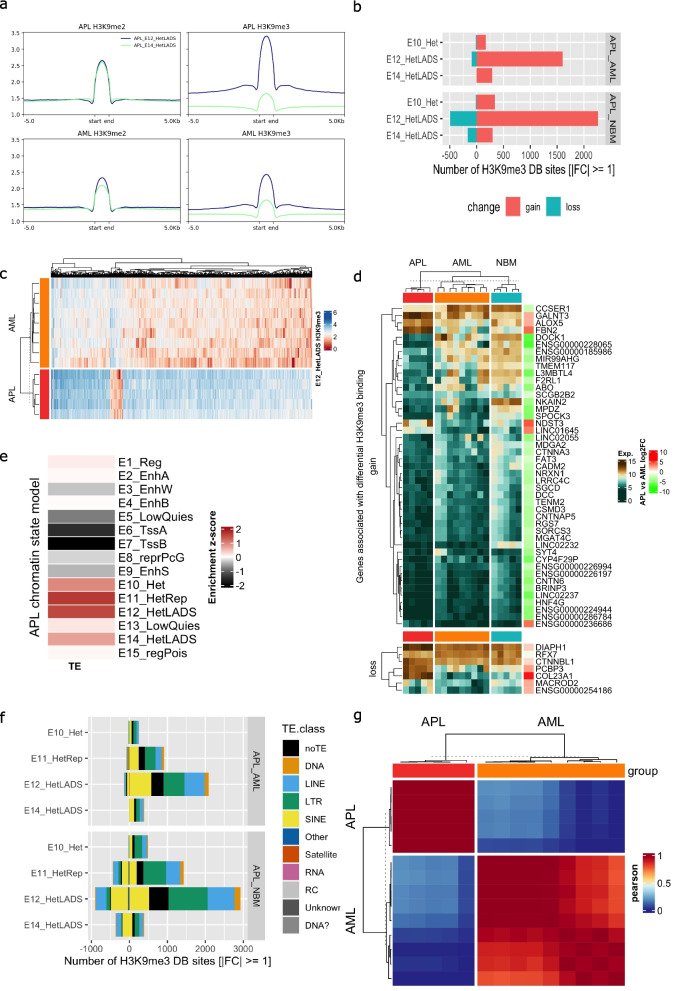
APL specific heterochromatin and transpons profiles.** a** Density plots of H3K9me2 and H3K9me3 profiles within lamina associated domain (LAD) related chromatin states E12_HetLADS and E14_HetLADS. **b** Bar plot showing gains and losses of H3K9me3 in heterochromatin-related chromatin states E10_Het, E11_HetRep, E12_HetLADS, and E14_HetLADS. **c** Heatmap of H3K9me3 modification profiles within E12_HetLADS in APL and AML patient cells. **d** Expression heatmap of genes with gain and loss of H3K9me3. **e** Enrichment of transposable elements (TE) (retrieved from Tetranscripts [67]) in the 15-chromatin state APL model. **f** Bar plot of TE classes distribution overlapping with sites with gain and loss of H3K9me3. **g** Heatmap of Pearson correlation of unsupervised clustering in Polycomb Group (PcG) gene-related chromatin state E8 in APL and AML samples

APL was characterized by major gains of H3K9me3 compared to both AML and normal bone marrow. This was found for all three analyzed heterochromatin states separating APL from AML (E10, E12, and E14), but with the greatest gain of H3K9me3 within the LAD associated state E12 (Fig. 5b). In contrast, there were no major differences in H3K9me3 modification between AML and NBM (Fig. S6a), suggesting that this disturbed H3K9me3 regulation was specific to APL cells. While APL samples homogeneously gained H3K9me3 modification in the E12 heterochromatin LADS state, AML samples showed a significantly more heterogeneous H3K9me3 pattern (Fig. 5c). Of the 707 genes that gained H3K9me3 in E12, only 45 were significantly differentially expressed in APL vs. AML of which 39 were downregulated in APL (Fig. 5d), consistent with the repressive effect of H3K9me3 modification.

As an important function of heterochromatin is to repress transposable elements (TE), we further investigated the changes of H3K9me3 within TEs, as defined by TE transcripts [67]. TEs were mostly enriched in the four heterochromatin related states E10, E11, E12, and E14 (Fig. 5e) and approximately 70% of the gained H3K9me3 sites within the heterochromatin states overlapped with TEs, mainly LINE, LTR, and SINE (Fig. 5f). Among TE subfamilies, Alu, ERV1, and ERVK, reported to play a role in genome folding [68], were the subfamilies that were most commonly targeted by change in H3K9me3 (Fig. S6b).

### HOX genes were repressed in APL with gained H3K27me3

As shown in the Pearson correlation heatmap (Fig. 5g), H3K27me3 in the polycomb associated state E8 could also completely separate APL from AML. Differential analysis of H3K27me3 within the E8 chromatin state was performed between APL and AML primary patient cells, revealing 5186 sites that gained and 3650 sites that lost H3K27me3 in APL. As expected, gained and lost H3K27me3 modification associated with decreased and increased gene expression, respectively (Fig. S6c). Genes in the HOX-gene regulatory network such as *HOXA3*,* HOXA9*,* MEIS1*, and *HOXB-AS3* were among the most downregulated genes that also gained H3K27me3 when comparing APL and AML (Fig. S6c). Also genes in the Reactome pathway “NRAGE signals death through JNK,” that is involved in apoptosis [69], showed the most significant change in H3K27me3. Thus, the change in gene expression associated with deregulated H3K27me3 may also contribute to leukemic growth in AML. The genes with gained H3K27me3 were predominantly upregulated in ATRA-treated NB4 cells, again especially during longer ATRA exposures (72 h and 120 h) (Fig. S6d).

## Discussion

This study provides a comprehensive analysis of the epigenetic landscape characterizing APL and its distinction from non-APL AML, by integrating multi-dimensional histone modification profiling and transcriptomics. PML-RARA contribute to leukemogenesis mainly via two main mechanisms: perturbing transcriptional regulation and disrupting *PML* nuclear bodies (NBs) [70, 71]. This study shows both direct and indirect deregulation of the epigenetic machinery, reflecting the two mechanistic functions of PML-RARA and its impact on epigenetic regulation. We uncovered unique chromatin state configurations and enhancer-driven transcriptional programs specific to APL. These findings offer novel insights into the pathogenesis of APL and identify key regulatory elements.

Our analysis identified distinct chromatin states in APL compared to AML and healthy samples. Chromatin states enriched for histone modifications associated with active transcription (e.g., H3K27ac, H3K4me1, and H3K18ac) in active enhancers (state E2) and active transcription start sites (TSS, state E6) were particularly prominent in APL. This distinct epigenetic signature is in stark contrast to the relatively heterogeneous histone profiles observed in AML.

The unsupervised clustering of histone modification profiles revealed that active enhancer and bivalent TSS chromatin states (e.g., E2, E4, E7) effectively separated APL from AML, suggesting that enhancer activity is central to the epigenetic identity of APL. However, only H3K27ac separate APL from AML and not the other enhancer histone marks, such as H3Kme1. Furthermore, the similarity of TSS-associated states between APL and AML contrasts to enhancer-associated states, in agreement with a previous study [9].

Previous studies have identified genome-wide PML-RARA binding sites in APL [8, 9, 25, 72, 73]. A striking observation was the strong enrichment of active histone marks at PML-RARA binding sites, particularly H3K27ac which is a marker of active enhancers. Despite the conventional view of PML-RARA as a dominant negative repressor, our findings suggest that PML-RARA fusion protein mainly localize to active enhancers, potentially functioning as a transcriptional activator at these regions. This duality underscores the complexity of PML-RARA’s role in APL pathogenesis and direct effects of PML-RARA. The t(15;17) not only gives rise to the PML-RARA fusion protein, but also changes the chromosomal structure at the break point. Our results suggest that promoters at chr17 hijack enhancers at chr15 and abnormally increase the expression of, e.g., ZNF385C while the exact role of this interaction should be studied further. For example, ATRA and arsenic treatment degrades PML-RAR and alleviates the differentiation block, despite the remained chromosomal translocation, suggesting that enhancer hijacking is not the primary driver of the APL associated differentiation block. However, it may still contribute leukemogenesis. In addition, PML-RARA binding in the vicinity of the identified super-enhancer region may enable the enhancer hijacking, suggesting that both PML-RAR and the genetic translocation interact to drive leukemogenesis. Similar mechanism of enhancer hijacking has been suggested for other chromosomal rearrangements, such as *ETV6::ACSL6* [74] and *MNX1::ETV6* [75]. Hence, t(15;17) deregulates enhancer activity via different mechanisms.

The identification of 6227 APL-specific enhancers, many of which are directly bound by PML-RARA, highlights the enhancer-centric regulatory landscape of APL. These enhancers, associated with the upregulation of genes implicated in hematopoietic differentiation and leukemogenesis, suggest that PML-RARA-driven enhancer activity plays a pivotal role in APL biology, underscoring the importance of enhancer regulation in driving leukemia-specific transcriptional programs. Recently, it has been demonstrated that induction of PML-RARA expression modulates the chromatin interactions that impacts gene expression [9], suggesting a role for deregulated enhancer usage in APL in agreement with our results. Inaccurately activated enhancers induce expression of genes that may contribute to the differentiation block in APL. UNCX and PPARG are examples of genes that are activated by the dysregulated enhancer activity in APL and may contribute to the differentiation block. UNCX is a transcription factor that has been suggested to be able to inhibit myeloid differentiation [48] and PPARG has been described to regulate myeloid differentiation [76, 77] and hematopoietic stress response [78].

Super enhancers, a subset of enhancers with exceptional regulatory potential, were found to be APL-specific and were associated with genes critical to APL pathogenesis. For example, the *INSR* (insulin receptor gene) and the small GTPase gene *RAB27A* were linked to super enhancers enriched within PML-RARA binding sites with chromatin interactions that were PML-RARA dependent. These genes exhibited robust upregulation in APL and have been implicated in cellular proliferation and signaling pathways, such as PI3K/MAPK/ERK, which are frequently deregulated in cancer. In addition, PML-RARA binding at super enhancers has recently been suggested to activate genes that contribute to leukemogenesis in APL [8], supporting our findings.

The presence of APL-specific super enhancers around key genes not only delineates the transcriptional landscape of APL but also reveals potential therapeutic vulnerabilities. The dependency of NB4 cells on APL super enhancer-associated genes, as shown through DepMap analysis, further underscores their functional importance for APL cell survival. Notably, ATRA-induced downregulation of *INSR*, a super enhancer-associated gene, highlights the potential therapeutic modulation of oncogenic enhancers by differentiation therapy*. INSR* is activating PI3K, MAPK, and ERK signaling and has been linked to several tumors including leukemia [79, 80] and *PPARG* can bind to *AP2-ɑ* and *SP1* and decrease the upregulation effect of *AP2-ɑ* on *INSR* in breast cancer [80–83].

Beyond enhancer regulation, H3K9me3 in heterochromatin-associated states (e.g., E10, E12, E14) also displayed significant differences between APL and AML. The ability of H3K9me3 to distinguish APL from AML is chromatin context dependent, since H3K9me3 does not separate APL and AML in the heterochromatin E11 that are enriched for repeats. The E10, E12, and E14 states are enriched for lamina-associated domains (LADs), which are critical for maintaining nuclear architecture and transcriptional repression. The differential organization of LADs may reflect broader changes in nuclear organization associated with APL pathogenesis.

It was recently shown that PML bodies can regulate H3K9me3 at the TE families Line-1 and *ERV*, via SUMOylation of *KAP1 *[84]. SUMOylation may control proteasomal degradation via recruitment of SUMO-targeted ubiquitin ligases [85]. PML body-mediated SUMOylation of *KAP1* has been suggested to regulate protein stability, but also protein interactions with *HP1* and the *SETDB1* methyltransferase causing H3K9me3 [86–88]. PML nuclear bodies (NB) are disrupted in PML-RARA-driven APL [89], which may disturb the PML-sumoylation-H3K9me3 axis and consequently perturb H3K9me3 at *KAP1* TE target families. In addition, also the catalytic Polycomb complex 2 member *EZH2* has been shown to interact with PML bodies [84] and may also be regulated via SUMOylation. We found that also the *EZH2* target H3K27me3 is deregulated in APL and can distinguish APL from AML.

While this study was not designed to assess the epigenetic landscape of non-APL AML, the analyses revealed a considerably more heterogenous epigenetic landscape in AML with less dominant heterochromatin marks as compared to APL.

*MEIS* and several HOX-genes can drive leukemia development and are known to be overexpressed in AML [90]. However, these genes are selectively downregulated in APL [91] and our data provide evidence of an APL-specific epigenetic pattern regulating MEIS as well as several of the HOX-genes. MEIS as well as HOXA3 and HOXA9 were to be among the most downregulated genes that also gained H3K27me3 and in addition to the gain of H3K27me3 these genes also lost active marks H3K4me3 and H3K27ac. While the expression patterns of these genes are well known in different subtypes of AML, we can here for the first time show the epigenetic patterns behind the APL-specific expression which also displays the robustness of the epigenetic approach.

## Conclusions

This study underscores the enhancer-driven transcriptional landscape that defines APL, where changes are targeted by binding of the PML-RARA fusion protein and where enhancer activation shape the disease-specific epigenome. The interplay between enhancers, super enhancers, and chromatin interactions offers novel insights into APL biology which can constitute a proof-of-concept of how different subtypes of AML are formed by unique chromatin structures. Future studies focusing on the functional validation of these enhancers and their associated genes will be critical in translating these findings into clinical applications.

## Supplementary Information

Additional file 1: Supplementary figures Fig. S1-S6. Fig. S1. Gene expression patterns and chromatin states in APL versus AML, and enrichment of ATRA-responsive genes around TSS regions. Fig. S2. Unsupervised consensus clustering based on the top 2000 most variable histone profiles within each chromatin state. Fig. S3. Unsupervised consensus clustering based on 2000 to 4000 of the most variable histone profiles within each chromatin state. Fig. S4. Characterization of PML-RARA–bound APL-specific enhancers through genomic annotation, chromatin state clustering, enhancer activity and correlation with gene expression, highlighting their functional enrichment and differential regulation in APL vs. AML and in ATRA-treated NB4 cells. Fig. S5. Differential expression of INSR and enhancer-associated genes during hematopoietic differentiation and ATRA treatment illustrating APL-specific super-enhancers and PML-RARA binding linked to altered regulation of S100P, LINC02481 and ZNF385C compared with AML and normal cells. Fig. S6. Gains and losses of H3K9me3 and H3K27me3 in heterochromatin states and their overlap with transposable elements as well as associated gene expression changes in AML and ATRA-treated NB4 cells.

Additional file 2: Table S1. Mutations in APL and AML cases and cytogenetics of APL cases. Table S2. Results from MEME motif analysis of APL-specific enhancer regions. Table S3. Gene Ontology enrichment based on motifs identified in APL-specific enhancer regions. Table S4. Correlation between ChromHMM-defined enhancers and gene expression. Table S5. Functional enrichment (KEGG, Reactome, Hallmark) of genes significantly associated with ChromHMM-defined enhancers. Table S6. Genes significantly associated with APL-specific super-enhancers.

## Data Availability

Due to Swedish Law and local regulations, raw sequencing data (HiC, ChIP-seq and RNA-seq of patient primary samples) cannot be shared on a public database. However, based on the ethical permission and applying Swedish Law and local regulations, the full raw data can be shared through a Data Transfer Agreement (DTA) directly between the university of the researchers and the university of a third partner. Depending on DTA process, the estimated time frame for sharing this data is 1 to 3 months. Researchers can get full access to the sequencing data through contact with the corresponding author, Sören Lehmann (soren.lehmann@ki.se). Processed data and cell line data are available at Zenodo [92] (10.5281/zenodo.17296520). The RNA-seq sequencing data have been deposited in NCBI GEO under accession number GSE305480 [93]. Publicly available datasets were used in this study, including PML-RARA binding sites from Tan *et al.* [8]; ChIA-PET data from Wang *et al.* [25]; gene expression profiles of AML and APL patients from AML ClinSeq cohort [27]; transposable element annotations from Tetranscripts [67], gene expression profiles of NB4 cells treated with ATRA from GEO by accession number GSE131325 [58], and gene expression data of normal hematopoietic differentiation retrieved from GEO by accession number GSE42519 [94]. Codes are available on github [95]: https://github.com/joey0214/Zhong_etal_APL_epigenetic_2025.

## Abbreviations

APL: Acute promyelocytic leukemia
AML: Acute myeloid leukemia
ATRA: All-trans-retinoic acid
ATO: Arsenic trioxide
NBM: Normal bone marrow
ChIP-seq: Chromatin immunoprecipitation followed by sequencing
TSS: Transcription start sites
GEO: Gene Expression Omnibus
LAD: Lamina-associated domain
RPKM: Fragments Per Kilobase Million
PcG: Polycomb Group
TF: Transcription factor
ROSE: Rank order of super enhancers
