# Supplementary material for: Disease-specific epigenetic deregulation of enhancers, transposons, and polycomb targets in acute promyelocytic leukemia
Source: Genome Med. 2025 Oct 30;17:135. doi: 10.1186/s13073-025-01565-y (PMC12573822; doi:10.1186/s13073-025-01565-y)
Supplement: Supplementary file 1 — Additional file 1: Supplementary figures Fig. S1-S6. Fig. S1. Gene expression patterns and chromatin states in APL versus AML, and enrichment of ATRA-responsive genes around TSS regions. Fig. S2. Unsupervised consensus clustering based on the top 2000 most variable histone profiles within each chromatin state. Fig. S3. Unsupervised consensus clustering based on 2000 to 4000 of the most variable histone profiles within each chromatin state. Fig. S4. Characterization of PML-RARA–bound APL-specific enhancers through genomic annotation, chromatin state clustering, enhancer activity and correlation with gene expression, highlighting their functional enrichment and differential regulation in APL vs. AML and in ATRA-treated NB4 cells. Fig. S5. Differential expression of INSR and enhancer-associated genes during hematopoietic differentiation and ATRA treatment illustrating APL-specific super-enhancers and PML-RARA binding linked to altered regulation of S100P, LINC02481 and ZNF385C compared with AML and normal cells. Fig. S6. Gains and losses of H3K9me3 and H3K27me3 in heterochromatin states and their overlap with transposable elements as well as associated gene expression changes in AML and ATRA-treated NB4 cells. [file 13073_2025_1565_MOESM1_ESM.pdf]

SUPPLEMENTARY INFORMATION

Disease specific epigenetic deregulation of enhancers, transposons and polycomb targets in acute promyelocytic leukemia

Xiangfu Zhong[1, *], Lina Cordeddu[2], Angelica Gamboa-Cedeno[3], Sofia Bengtzén[1], Karl Ekwall[2], Andreas Lennartsson[1, *,#], Sören Lehmann[1,3, *,#]

Supplementary figures

Fig. S1. a Density plot of gene expression levels in APL and AML patient samples as defined as high (RPKM >2), and low (RPKM < 0.01) expression. **b** Functional genomic annotation enrichment using publicly available data for the 15 chromatin states in the AML patient group. **c** Heatmap of Jaccard index of chromatin states between APL and AML. **d** Enrichment of ATRA responsive genes in the 15 APL chromatin states. TSS regions are defined as TSS ±500bp, OverlapEnrichment of ATRA responsive genes (UP/DOWN regulated in ATRA treatment vs. control in NB4 cells).

Fig. S2. Unsupervised consensus clustering (from k=2 to k=5) based on the top 2000 most variable histone profiles within each chromatin state

Fig. S3. Unsupervised consensus clustering (from k=2 to k=5) based on 2000-4000 most variable histone profiles within each chromatin state showing no significant improvement compared to the top 2000 most variable histone profiles as shown in Supplementary Fig. 2.

Fig. S4. a Heat map of correlations of unsupervised clustering using the top 2000 most variable features of H3K27ac profiles within E2_EnhA chromatin state in APL and AML samples. **b** Genomic annotation of PML-RARA target sites. **c** Heat map of H3K27ac in AML-specific enhancers in APL and AML samples. **d** GO biological process enrichment of genes correlating to APL specific enhancers (Pearson correlation *p*-value below 0.01, correlation above 0.7). **e** The HALLMARK, KEGG and REACTOME enrichment of genes correlating to APL specific enhancers. **f** Bar plot of percentage of enhancer associated genes in differential expression analysis (LFC: log2FoldChange, green for not significant, blue for LFC < 1, purple for LFC >= 1). **g** The heatmap showing the log2FoldChange in expression (by RNA-seq) of genes associated to APL enhancers in APL vs. AML patient samples and in NB4 cells exposed to ATRA vs. control RNA-seq. **h** Bar plot of the number of genes associated with APL specific and PML-RARA bound enhancers differential expressed in ATRA treatment NB4 cells from GSE131325.

Fig. S5. a Scatter plot of *INSR* expression during normal hematopoietic differentiation (GSE42519), t-test performed for comparing early and late promyelocytes with hematopoietic stem cells. **b** Scatter plot of *INSR* expression in NB4 cells exposed to ATRA or DMSO during different time points (GSE131325). p.adj=0.0003, log2FoldChange = -1.5. **c**

INSR expression in NB4 cells exposed to ATRA or DMSO in this study. **d** Bar plot of the number of genes associated with APL specific and PML-RARA bound super-enhancers differential expressed in ATRA treatment NB4 cells from GSE131325. **e** IGV track of *S100P* showing APL specific super enhancers, APL-specific enhancers, PML-RARA binding sites, H3K27ac profile in APL, H3K27ac profile in AML, H3K4me3 profile in APL, H3K4me3 profile in AML, RNAPOLⅡ loops by ChIA-PET in U937 cells (without PML-RARA expression), RNAPOLⅡ loops by ChIA-PET in U937+$ZnSO_4$ cells (with PML-RARA expression). **f** and **g** Relative expression of *S100P* (**f**) and *LINC02481* (**g**) in APL and AML patient cells and normal $CD34^+$ bone marrow cells. **h** IGV track of zoomed region at chr17 (data displayed as described above for panel **d**). **i** Box plot of *ZNF385C* in APL and AML patient cells and normal $CD34^+$ bone marrow cells.

Fig. S6. a Bar plot showing gains and losses of H3K9me3 in heterochromatin-related chromatin states E10_Het, E11_HetRep, E12_HetLADS and E14_HetLADS, including AML vs NBM. **b** Bar plot showing the distribution of transposable element families that overlaps with gain and loss of H3K9me3. **c** Expression heatmap of genes associated with significant gain (right) and loss (left) H3K9me3 modification within the E14_HetLADS state. **d** Bar plot of the number of genes with gained H3K27me3 differential expressed in ATRA treatment NB4 cells from GSE131325.

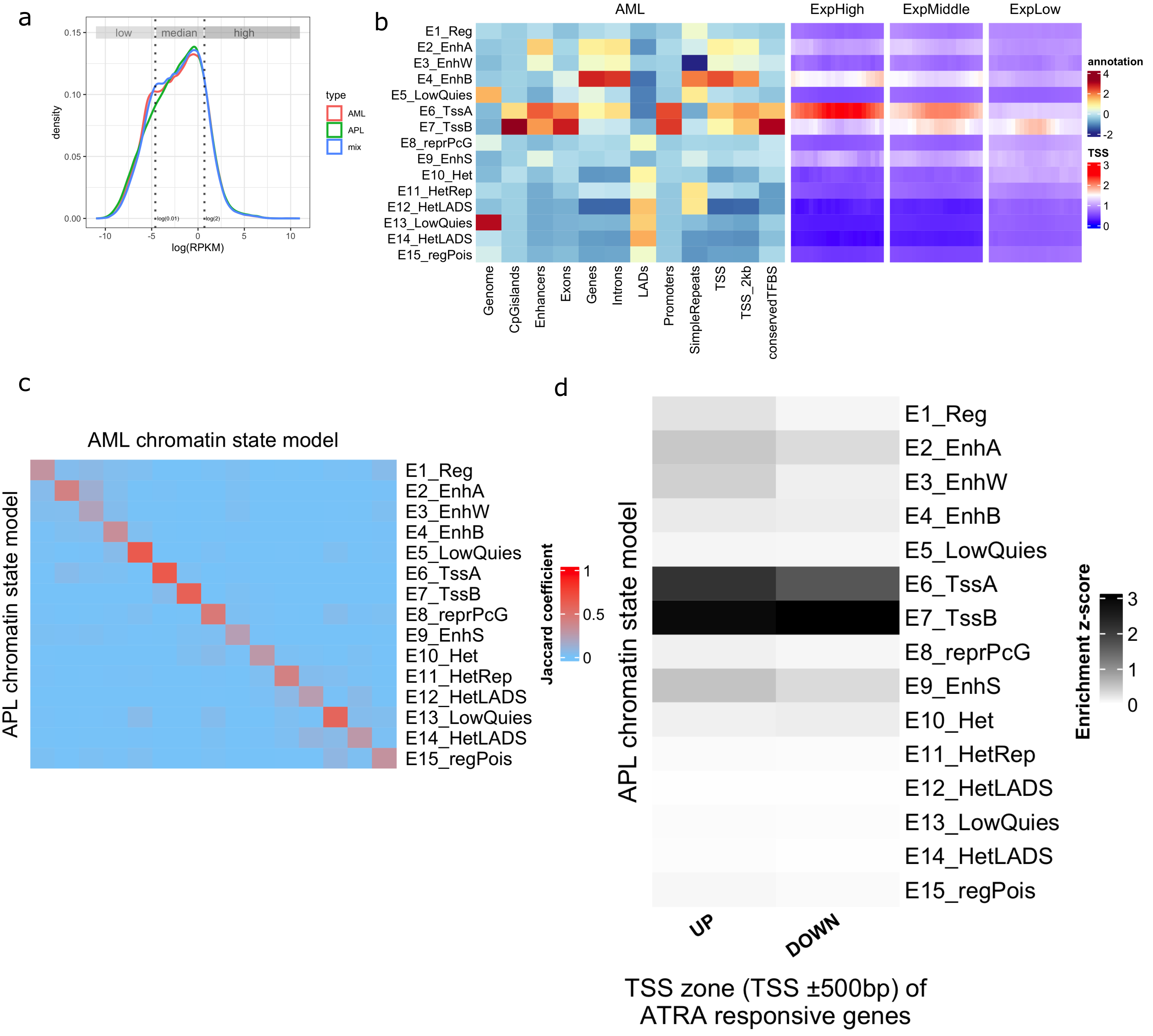

a
low
median
high
density
log(RPKM)
type
AML
APL
mix
b
AML
ExpHigh
ExpMiddle
ExpLow
annotation
TSS
E1_Reg
E2_EnhA
E3_EnhW
E4_EnhB
E5_LowQuies
E6_TssA
E7_TssB
E8_reprPcG
E9_EnhS
E10_Het
E11_HetRep
E12_HetLADS
E13_LowQuies
E14_HetLADS
E15_regPois
Genome
CpGislands
Enhancers
Exons
Genes
Introns
LADs
Promoters
SimpleRepeats
TSS
TSS_2kb
conservedTFBS
c
AML chromatin state model
APL chromatin state model
Jaccard coefficient
d
APL chromatin state model
Enrichment z-score
UP
DOWN
TSS zone (TSS ±500bp) of
ATRA responsive genes

Fig. S1

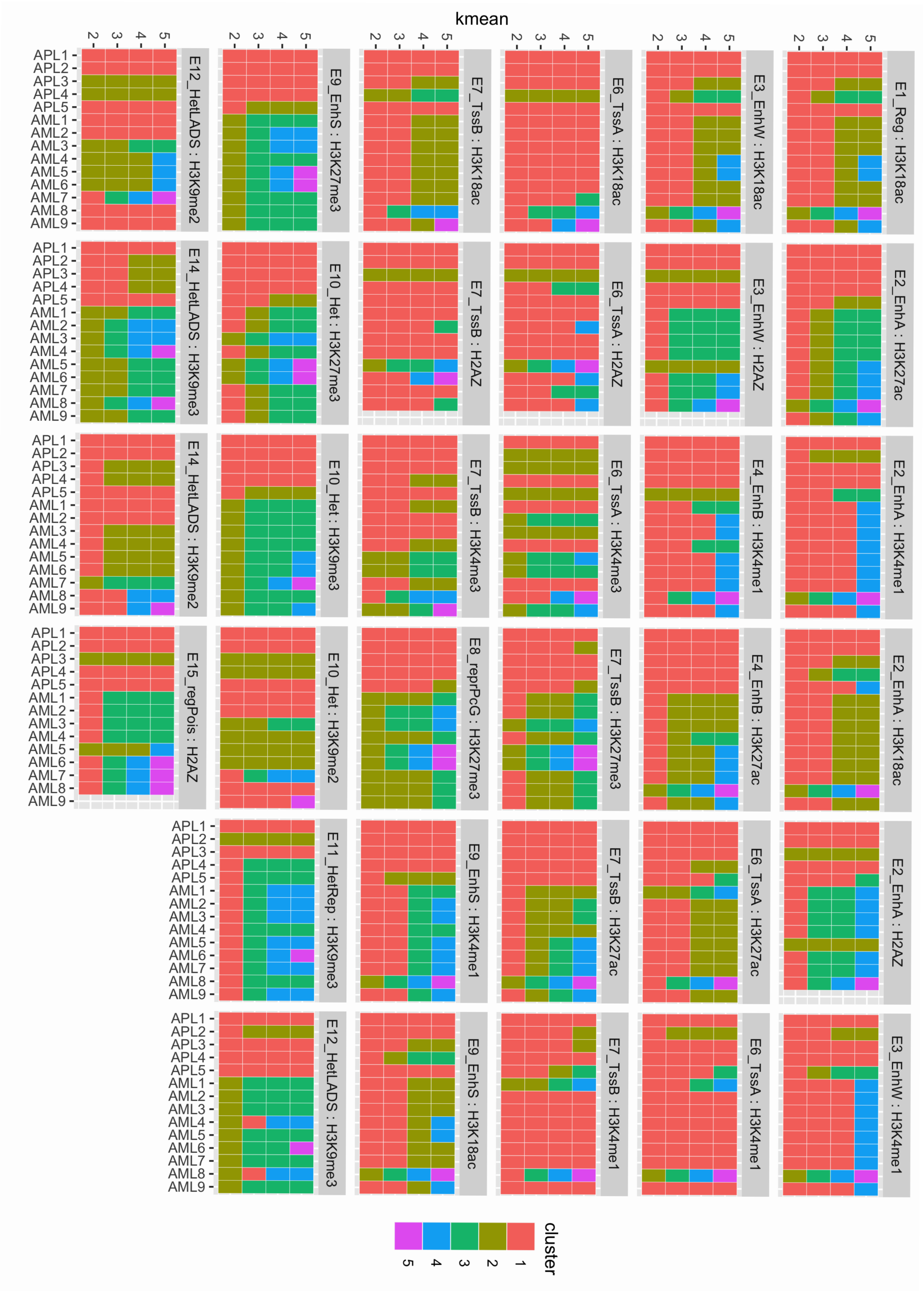

kmean
2
3
4
5
APL1
APL2
APL3
APL4
APL5
AML1
AML2
AML3
AML4
AML5
AML6
AML7
AML8
AML9
E12_HetLADS : H3K9me2
E9_EnhS : H3K27me3
E7_TssB : H3K18ac
E6_TssA : H3K18ac
E3_EnhW : H3K18ac
E1_Reg : H3K18ac
E14_HetLADS : H3K9me3
E10_Het : H3K27me3
E7_TssB : H2AZ
E6_TssA : H2AZ
E3_EnhW : H2AZ
E2_EnhA : H3K27ac
E14_HetLADS : H3K9me2
E10_Het : H3K9me3
E7_TssB : H3K4me3
E6_TssA : H3K4me3
E4_EnhB : H3K4me1
E2_EnhA : H3K4me1
E15_regPois : H2AZ
E10_Het : H3K9me2
E8_reprPcG : H3K27me3
E7_TssB : H3K27me3
E4_EnhB : H3K27ac
E2_EnhA : H3K18ac
E11_HetRep : H3K9me3
E9_EnhS : H3K4me1
E7_TssB : H3K27ac
E6_TssA : H3K27ac
E2_EnhA : H2AZ
E12_HetLADS : H3K9me3
E9_EnhS : H3K18ac
E7_TssB : H3K4me1
E6_TssA : H3K4me1
E3_EnhW : H3K4me1
cluster
1
2
3
4
5

Fig. S2

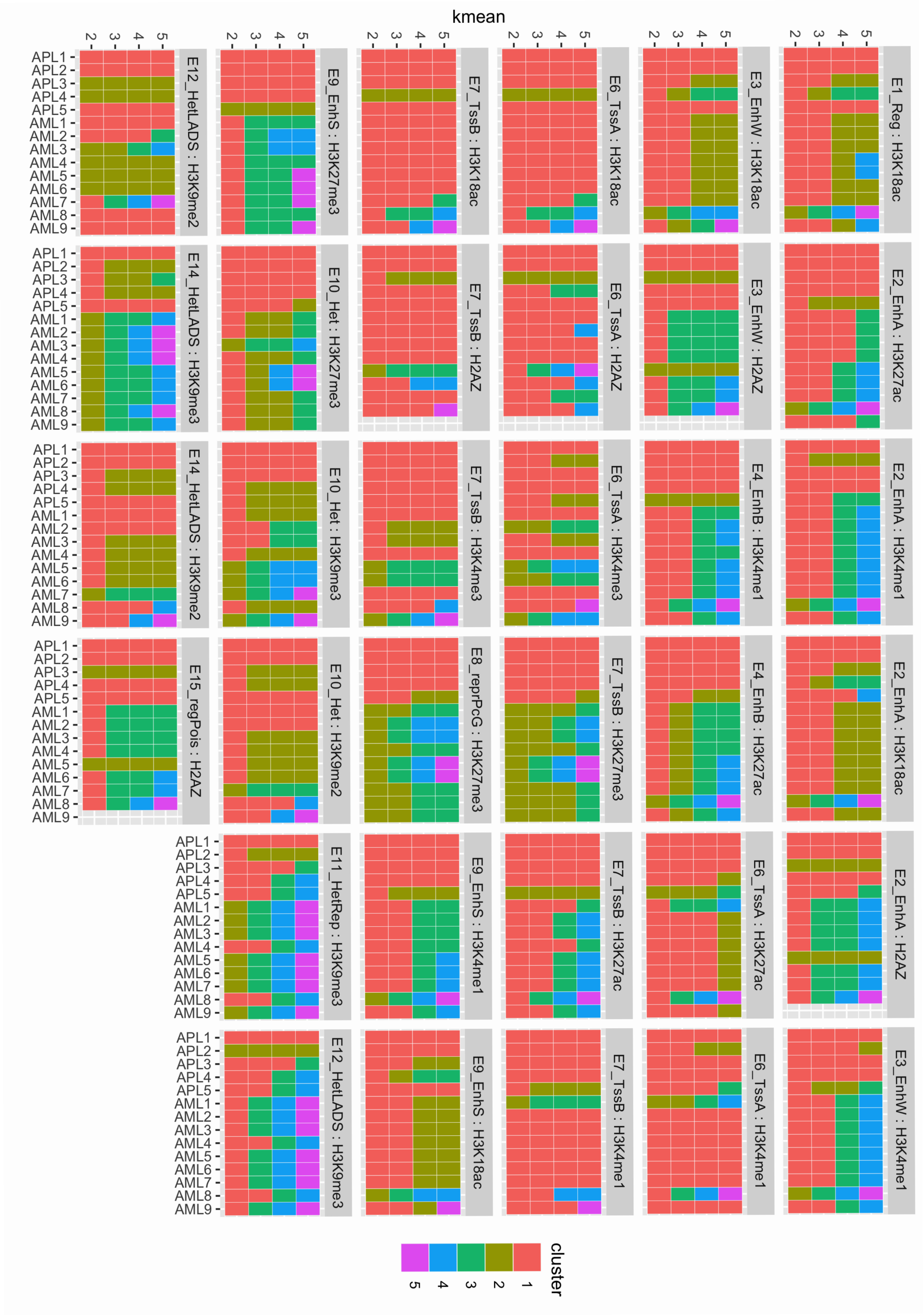
kmean
2
3
4
5
APL1
APL2
APL3
APL4
APL5
AML1
AML2
AML3
AML4
AML5
AML6
AML7
AML8
AML9
E12_HetLADS : H3K9me2
E9_EnhS : H3K27me3
E7_TssB : H3K18ac
E6_TssA : H3K18ac
E3_EnhW : H3K18ac
E1_Reg : H3K18ac
E14_HetLADS : H3K9me3
E10_Het : H3K27me3
E7_TssB : H2AZ
E6_TssA : H2AZ
E3_EnhW : H2AZ
E2_EnhA : H3K27ac
E14_HetLADS : H3K9me2
E10_Het : H3K9me3
E7_TssB : H3K4me3
E6_TssA : H3K4me3
E4_EnhB : H3K4me1
E2_EnhA : H3K4me1
E15_regPois : H2AZ
E10_Het : H3K9me2
E8_reprPcG : H3K27me3
E7_TssB : H3K27me3
E4_EnhB : H3K27ac
E2_EnhA : H3K18ac
E11_HetRep : H3K9me3
E9_EnhS : H3K4me1
E7_TssB : H3K27ac
E6_TssA : H3K27ac
E2_EnhA : H2AZ
E12_HetLADS : H3K9me3
E9_EnhS : H3K18ac
E7_TssB : H3K4me1
E6_TssA : H3K4me1
E3_EnhW : H3K4me1
cluster
1
2
3
4
5

Fig. S3

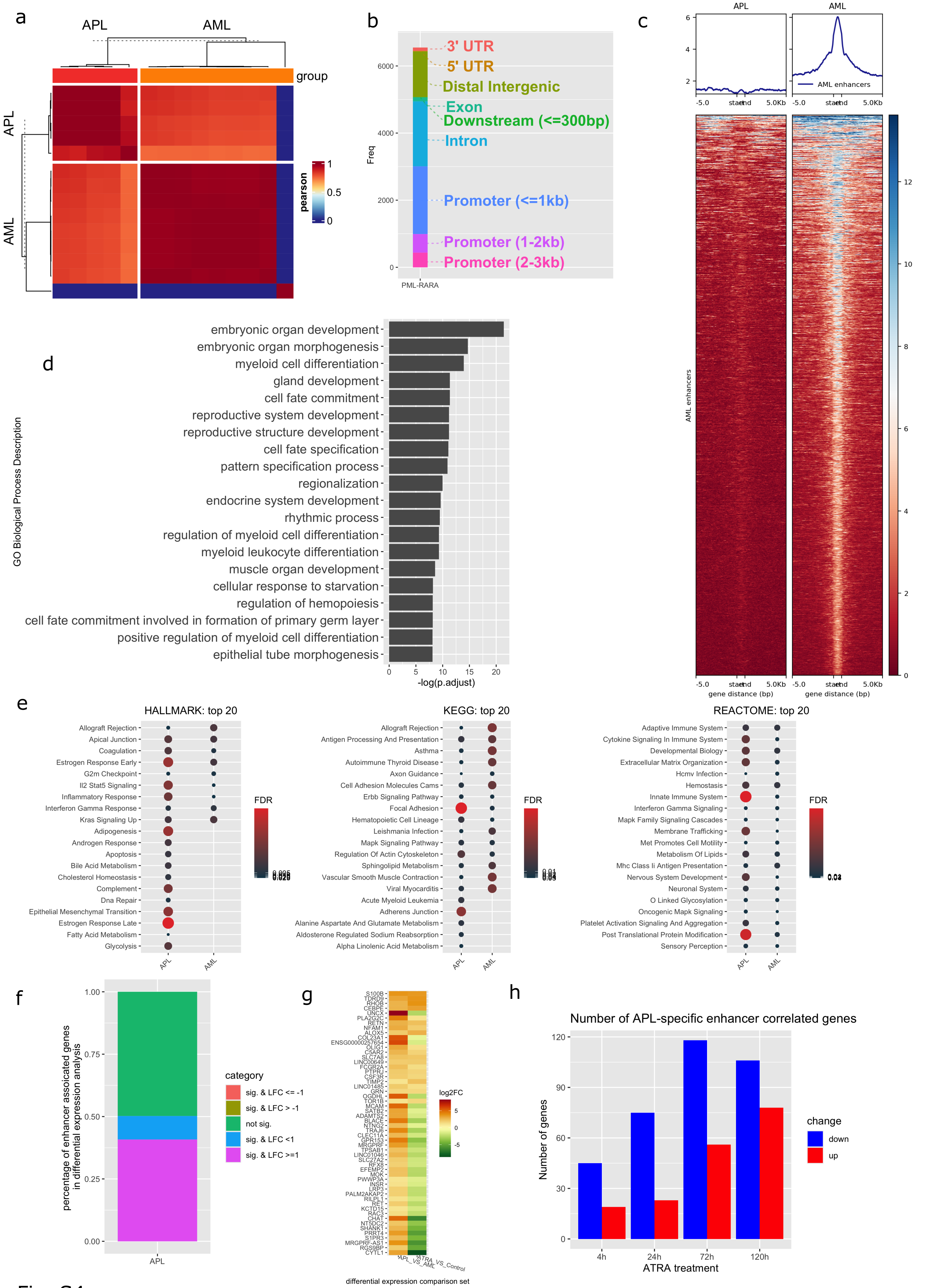
a
APL
AML
group
pearson
1
0.5
0
b
3' UTR
5' UTR
Distal Intergenic
Exon
Downstream (<=300bp)
Intron
Promoter (<=1kb)
Promoter (1-2kb)
Promoter (2-3kb)
Freq
6000
4000
2000
0
PML-RARA
c
APL
AML
AML enhancers
-5.0
start
end
5.0Kb
gene distance (bp)
12
10
8
6
4
2
0
d
GO Biological Process Description
embryonic organ development
embryonic organ morphogenesis
myeloid cell differentiation
gland development
cell fate commitment
reproductive system development
reproductive structure development
cell fate specification
pattern specification process
regionalization
endocrine system development
rhythmic process
regulation of myeloid cell differentiation
myeloid leukocyte differentiation
muscle organ development
cellular response to starvation
regulation of hemopoiesis
cell fate commitment involved in formation of primary germ layer
positive regulation of myeloid cell differentiation
epithelial tube morphogenesis
0
5
10
15
20
-log(p.adjust)
e
HALLMARK: top 20
Allograft Rejection
Apical Junction
Coagulation
Estrogen Response Early
G2m Checkpoint
Il2 Stat5 Signaling
Inflammatory Response
Interferon Gamma Response
Kras Signaling Up
Adipogenesis
Androgen Response
Apoptosis
Bile Acid Metabolism
Cholesterol Homeostasis
Complement
Dna Repair
Epithelial Mesenchymal Transition
Estrogen Response Late
Fatty Acid Metabolism
Glycolysis
KEGG: top 20
Allograft Rejection
Antigen Processing And Presentation
Asthma
Autoimmune Thyroid Disease
Axon Guidance
Cell Adhesion Molecules Cams
Erbb Signaling Pathway
Focal Adhesion
Hematopoietic Cell Lineage
Leishmania Infection
Mapk Signaling Pathway
Regulation Of Actin Cytoskeleton
Sphingolipid Metabolism
Vascular Smooth Muscle Contraction
Viral Myocarditis
Acute Myeloid Leukemia
Adherens Junction
Alanine Aspartate And Glutamate Metabolism
Aldosterone Regulated Sodium Reabsorption
Alpha Linolenic Acid Metabolism
REACTOME: top 20
Adaptive Immune System
Cytokine Signaling In Immune System
Developmental Biology
Extracellular Matrix Organization
Hcmv Infection
Hemostasis
Innate Immune System
Interferon Gamma Signaling
Mapk Family Signaling Cascades
Membrane Trafficking
Met Promotes Cell Motility
Metabolism Of Lipids
Mhc Class Ii Antigen Presentation
Nervous System Development
Neuronal System
O Linked Glycosylation
Oncogenic Mapk Signaling
Platelet Activation Signaling And Aggregation
Post Translational Protein Modification
Sensory Perception
FDR
APL
AML
f
percentage of enhancer assoicated genes in differential expression analysis
1.00
0.75
0.50
0.25
0.00
APL
category
sig. & LFC <= -1
sig. & LFC > -1
not sig.
sig. & LFC <1
sig. & LFC >=1
g
S100B
TDRD9
RHOB
CEBPE
UNCX
PLA2G2C
RETN
NFAM1
ALOX5
COL23A1
ENSG00000257654
OLIG1
C5AR2
SLC7A8
LINC00649
FCGR2A
PTPRJ
CSF3R
TIMP2
LINC01485
GRN
OGDHL
TOR1B
MCAM
SATB2
ADAMTS2
BLACE
NTNG2
TRAJ6
CLEC11A
GPR153
MRGPRF
TPSAB1
LINC01046
SLC27A2
RFX8
EFEMP2
MOK
PWWP3A
INSR
LRP3
PALM2AKAP2
RILPL1
RET
KCTD15
RAC3
CHAT
NT5DC2
SHANK1
PRRT4
S1PR3
MRGPRF-AS1
RGS9BP
CYTL1
APL_VS_AML
ATRA_VS_Control
differential expression comparison set
log2FC
5
0
-5
h
Number of APL-specific enhancer correlated genes
Number of genes
120
90
60
30
0
4h
24h
72h
120h
ATRA treatment
change
down
up

Fig. S4

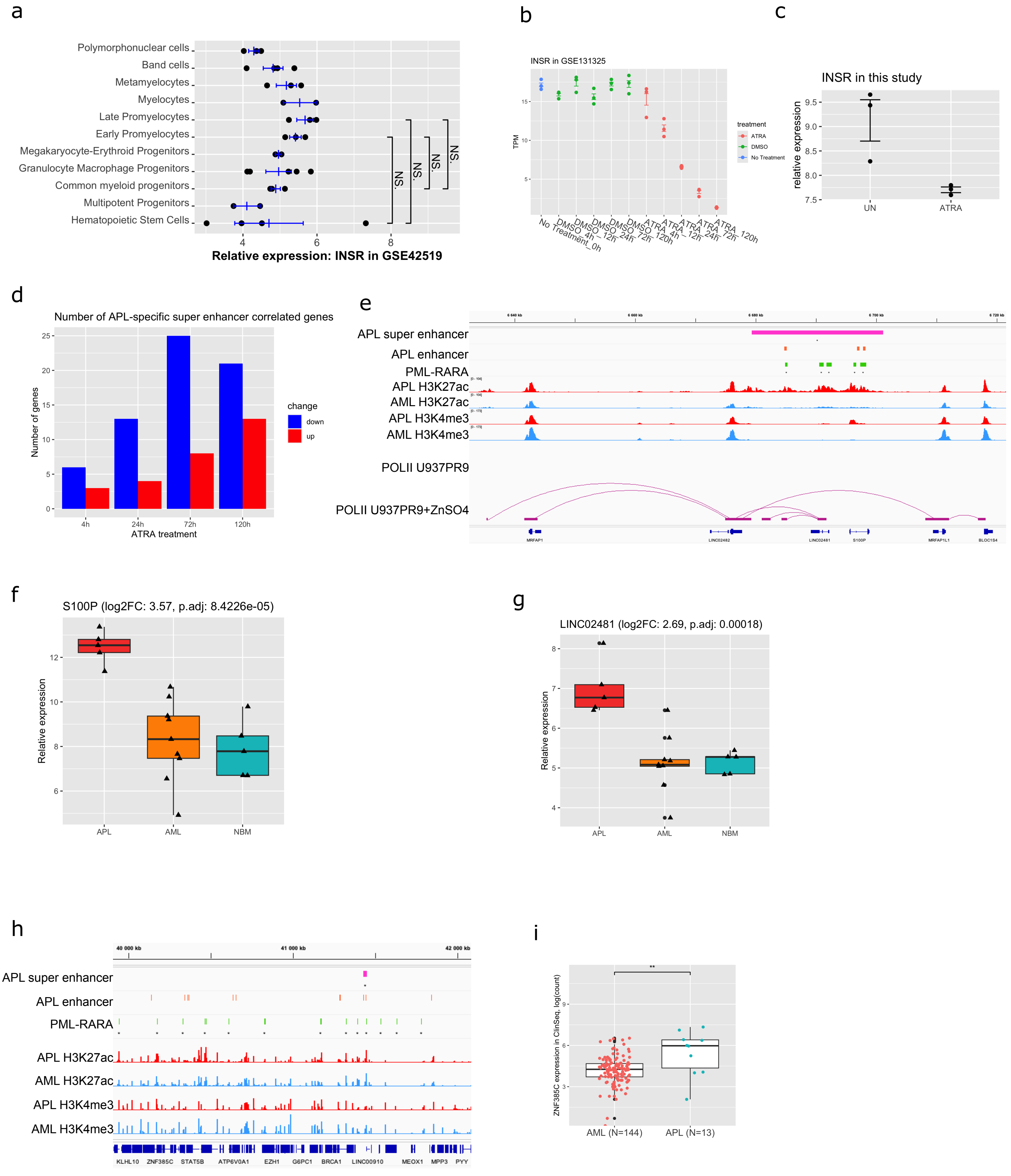

a
Polymorphonuclear cells
Band cells
Metamyelocytes
Myelocytes
Late Promyelocytes
Early Promyelocytes
Megakaryocyte-Erythroid Progenitors
Granulocyte Macrophage Progenitors
Common myeloid progenitors
Multipotent Progenitors
Hematopoietic Stem Cells
N.S.
N.S.
N.S.
N.S.
Relative expression: INSR in GSE42519
b
INSR in GSE131325
TPM
treatment
ATRA
DMSO
No Treatment
No Treatment_0h
DMSO_4h
DMSO_12h
DMSO_24h
DMSO_72h
DMSO_120h
ATRA_4h
ATRA_12h
ATRA_24h
ATRA_72h
ATRA_120h
c
INSR in this study
relative expression
UN
ATRA
d
Number of APL-specific super enhancer correlated genes
Number of genes
change
down
up
4h
24h
72h
120h
ATRA treatment
e
APL super enhancer
APL enhancer
PML-RARA
APL H3K27ac
AML H3K27ac
APL H3K4me3
AML H3K4me3
POLII U937PR9
POLII U937PR9+ZnSO4
MRFAP1
LINC02482
LINC02481
S100P
MRFAP1L1
BLOC1S4
f
S100P (log2FC: 3.57, p.adj: 8.4226e-05)
Relative expression
APL
AML
NBM
g
LINC02481 (log2FC: 2.69, p.adj: 0.00018)
Relative expression
APL
AML
NBM
h
40 000 kb
41 000 kb
42 000 kb
APL super enhancer
APL enhancer
PML-RARA
APL H3K27ac
AML H3K27ac
APL H3K4me3
AML H3K4me3
KLHL10
ZNF385C
STAT5B
ATP6V0A1
EZH1
G6PC1
BRCA1
LINC00910
MEOX1
MPP3
PYY
i
**
ZNF385C expression in ClinSeq, log(count)
AML (N=144)
APL (N=13)

Fig. S5

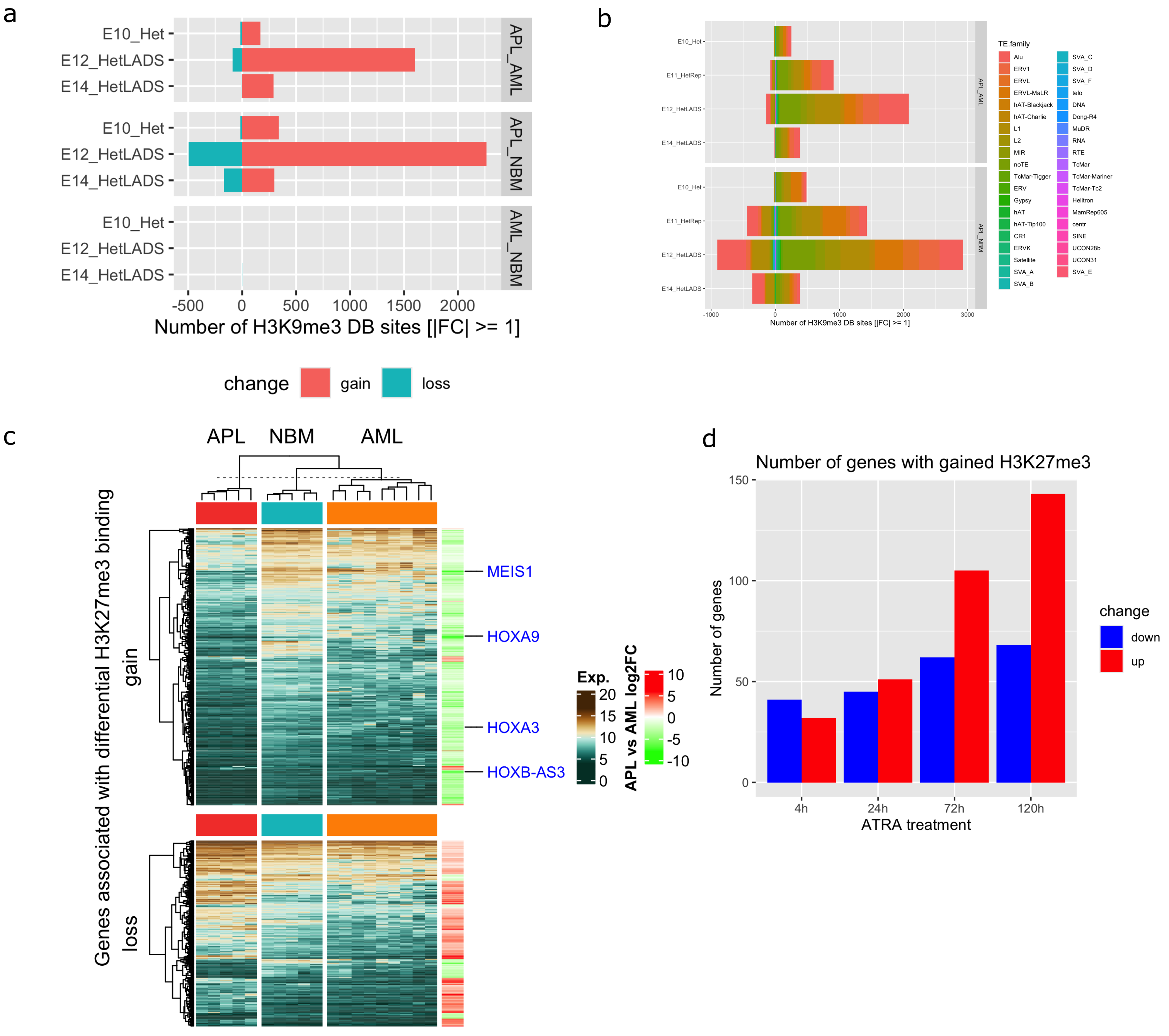

a
E10_Het
E12_HetLADS
E14_HetLADS
APL_AML
APL_NBM
AML_NBM
-500
0
500
1000
1500
2000
Number of H3K9me3 DB sites [|FC| >= 1]
change
gain
loss
b
E10_Het
E11_HetRep
E12_HetLADS
E14_HetLADS
APL_AML
APL_NBM
-1000
0
1000
2000
3000
Number of H3K9me3 DB sites [|FC| >= 1]
TE.family
Alu
ERV1
ERVL
ERVL-MaLR
hAT-Blackjack
hAT-Charlie
L1
L2
MIR
noTE
TcMar-Tigger
ERV
Gypsy
hAT
hAT-Tip100
CR1
ERVK
Satellite
SVA_A
SVA_B
SVA_C
SVA_D
SVA_F
telo
DNA
Dong-R4
MuDR
RNA
RTE
TcMar
TcMar-Mariner
TcMar-Tc2
Helitron
MamRep605
centr
SINE
UCON28b
UCON31
SVA_E
c
APL
NBM
AML
Genes associated with differential H3K27me3 binding
gain
loss
MEIS1
HOXA9
HOXA3
HOXB-AS3
Exp.
20
15
10
5
0
APL vs AML log2FC
10
5
0
-5
-10
d
Number of genes with gained H3K27me3
Number of genes
150
100
50
0
4h
24h
72h
120h
ATRA treatment
change
down
up

Fig. S6
